# Virtual Reality for Management of Pain in Hospitalized Patients: Results of a Controlled Trial

**DOI:** 10.2196/mental.7387

**Published:** 2017-03-29

**Authors:** Vartan C Tashjian, Sasan Mosadeghi, Amber R Howard, Mayra Lopez, Taylor Dupuy, Mark Reid, Bibiana Martinez, Shahzad Ahmed, Francis Dailey, Karen Robbins, Bradley Rosen, Garth Fuller, Itai Danovitch, Waguih IsHak, Brennan Spiegel

**Affiliations:** ^1^ Cedars-Sinai Medical Center Health Services Research Los Angeles,CA, CA United States

**Keywords:** pain, virtual reality, inpatients, hospitalization

## Abstract

**Background:**

Improvements in software and design and reduction in cost have made virtual reality (VR) a practical tool for immersive, three-dimensional (3D), multisensory experiences that distract patients from painful stimuli.

**Objective:**

The objective of the study was to measure the impact of a onetime 3D VR intervention versus a two-dimensional (2D) distraction video for pain in hospitalized patients.

**Methods:**

We conducted a comparative cohort study in a large, urban teaching hospital in medical inpatients with an average pain score of ≥3/10 from any cause. Patients with nausea, vomiting, dementia, motion sickness, stroke, seizure, and epilepsy and those placed in isolation were excluded. Patients in the intervention cohort viewed a 3D VR experience designed to reduce pain using the Samsung Gear Oculus VR headset; control patients viewed a high-definition, 2D nature video on a 14-inch bedside screen. Pre- and postintervention pain scores were recorded. Difference-in-difference scores and the proportion achieving a half standard deviation pain response were compared between groups.

**Results:**

There were 50 subjects per cohort (N=100). The mean pain reduction in the VR cohort was greater than in controls (−1.3 vs −0.6 points, respectively; *P*=.008). A total of 35 (65%) patients in the VR cohort achieved a pain response versus 40% of controls (*P*=.01; number needed to treat=4). No adverse events were reported from VR.

**Conclusions:**

Use of VR in hospitalized patients significantly reduces pain versus a control distraction condition. These results indicate that VR is an effective and safe adjunctive therapy for pain management in the acute inpatient setting; future randomized trials should confirm benefit with different visualizations and exposure periods.

**Trial Registration:**

Clinicaltrials.gov NCT02456987; https://clinicaltrials.gov/ct2/show/NCT02456987 (Archived by WebCite at http://www.webcitation.org/6pJ1P644S)

## Introduction

Hospitalized patients frequently experience physical, emotional, and social distress that is exacerbated by a radical change in living environment, loss of customary rights and privileges, and a high prevalence of pain [1]. Nearly half of hospitalized patients experience pain, of which a quarter is considered “unbearable” [2]. In order to care for the whole patient, hospital clinicians must consider not only the physical impact of illness, but also the psychosocial impact. However, the dynamic nature of hospital medicine, coupled with limited time to spend with individual patients, poses challenges to offering holistic inpatient care.

Treatment of pain in the acute care setting is often focused on pharmacological management, which can yield inconsistent and suboptimal pain control [3]. However, extensive data reveal that adjunctive nonpharmacological techniques, such as cognitive behavioral therapy and relaxation techniques, can modify cognitions and behaviors that influence the perception of pain [4].

Virtual reality (VR) technology provides an immersive, multisensory, and three-dimensional (3D) environment that enables users to have modified experiences of reality by creating a sense of “presence” [5,6]. To date, VR has been used in numerous clinical settings to help treat anxiety disorders, control pain, support physical rehabilitation, and distract patients during wound care [5-13]. For example, VR coupled with medication is effective in decreasing pain during bandage changes for severe burns [7,11,14,15]. Similarly, VR reduces pain and provides positive distraction during routine procedures such as intravenous line placements [10] and dental procedures [8,16]. Other studies reveal that VR helps manage chronic pain conditions such as complex regional pain syndrome [17] and chronic neck pain [18]. By stimulating the visual, auditory, and proprioception senses, VR acts as a distraction to limit the user’s processing of nociceptive stimuli [6].

However, the evidence to date supporting VR for inpatient care has shortcomings. In a recent meta-analysis of randomized controlled trials of VR for medical inpatients, we found 11 previous studies testing VR versus control conditions [19]. Although VR was effective and well tolerated in most studies, the trials were generally small, of variable methodological quality, limited to one indication at a time (eg, physical trauma, stroke rehabilitation, brain injury, cancer pain), and not focused on the acute care setting. It remains unclear if VR is superior to conventional means of pain distraction, such as viewing two-dimensional (2D) images, particularly in diverse populations of hospitalized, acute care patients suffering from varying types of pain. We have previously published data evaluating the feasibility and initial qualitative experience of using VR in hospitalized patients [20] but have not evaluated its impact on patient perception versus a control condition in hospitalized patients. In this study, we measured the impact of a 3D VR pain distraction experience versus a 2D pain distraction video in a diverse group of hospitalized patients with varying types of somatic and visceral pain.

## Methods

### Participants

We conducted a nonrandomized, comparative cohort study over a 6-month period to compare a 3D VR pain distraction experience (administered during the first 3-month recruitment period) with a 2D high-definition nature video on a 14-in screen placed in easy viewing proximity (administered during the second 3-month period), described further below. In both cohorts, we recruited adults (18+ years) admitted to the Inpatient Specialty Program at Cedars-Sinai Medical Center, a large, urban, tertiary care medical center. We excluded patients who could not consent, who were placed in contact isolation, or who had head wounds or bandages that interfere with the VR headset. In addition, because VR may cause motion sickness in some users [21], we excluded patients with a history of motion sickness and vertigo and anyone experiencing active nausea or vomiting. Patients with a history of seizures or epilepsy were also excluded to limit the theoretical risk of inducing seizures with VR (Samsung Gear user manual cites a 0.025% risk from pediatric data). Patients with an average pain score of ≥3 out of 10 during the 24 hours preceding patient screening were selected to participate in the study. We applied the same inclusion and exclusion criteria for both cohorts and approached all eligible patients in order of service admission.

### Interventions

#### Virtual Reality Pain Distraction Experience

**Figure 1:**
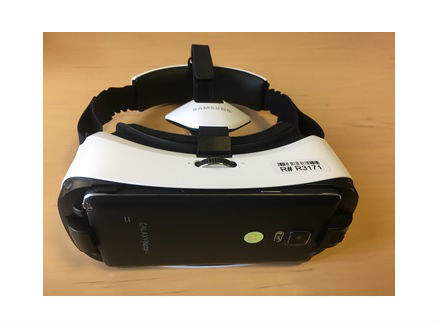
Samsung Gear virtual reality headset.

**Figure 2:**
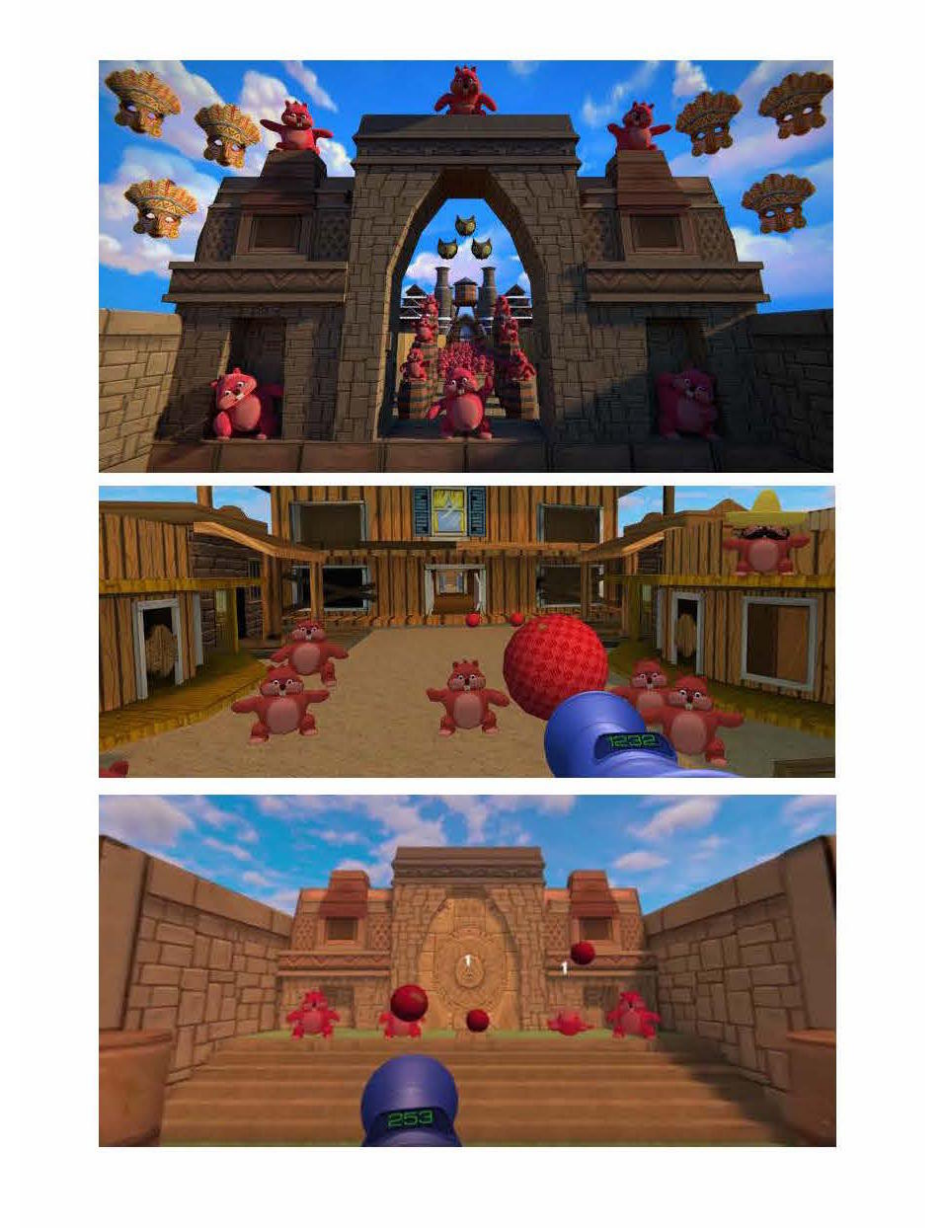
Screenshots of Pain RelieVR immersive pain distraction experience.

**Figure 3:**
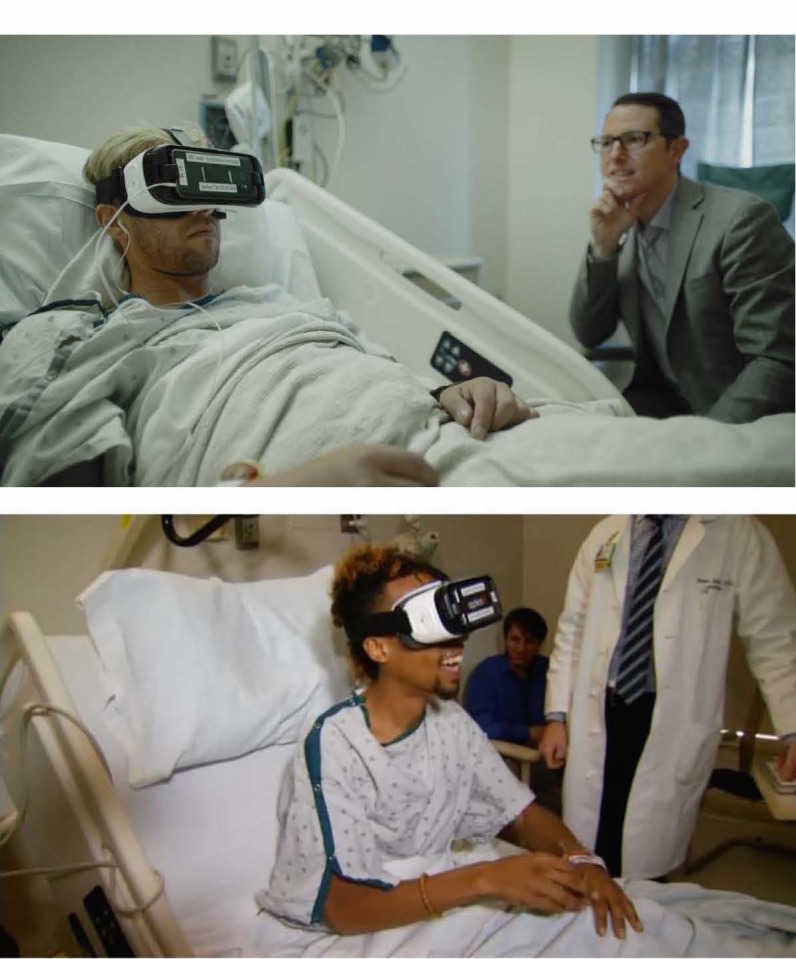
Hospitalized patients using Samsung Gear headsets (with written patient permission).

We administered VR using the Samsung (Ridgefield Park, NJ 07660) Gear Occulus headset fitted with a Samsung Galaxy S7 phone that delivers VR images and sound (Figure 1). We selected the Samsung Gear because it is commercially available, widely used, relatively inexpensive, has minimal visual latency, and offers a generally positive patient experience based on our previous research [20]. Higher-end tethered headsets, such as the Oculus Rift, are currently more expensive and onerous to use at scale in an inpatient setting. We used disposable sanitary covers and foam backing on each headset between patient uses and sanitized the equipment using the protocol we described in previous research [20]. Patients watched a 15-minute VR experience called Pain RelieVR, specifically designed to treat pain in patients who are bedbound or have limited mobility (AppliedVR, Los Angeles, CA, USA; Figure 2). Pain RelieVR is an immersive, 360-degree, game experience that takes place in a fantasy world where the user attempts to shoot balls at a wide range of moving objects by maneuvering his or her head toward the targets. This engaging, medium-intensity activity is free of interruption, offering the user a distracting experience designed to reduce the perception of pain. Pain RelieVR is a nonviolent and noncompetitive game that incorporates motivational music and features positively reinforcing sounds, animation, and direct messages to patients. Forward-facing action allows bedbound patients to engage without having to turn backward or contort into potentially uncomfortable positions. Figure 3 shows example patients using the Samsung Gear headsets (used with written patient permission).

#### Two-Dimensional Pain Distraction Experience

During the control period, we administered a 2D high-definition (1080p) video depicting relaxing nature scenes, including mountain lakes and running streams from Patagonian vistas presented with an audio track featuring Native American Shaman music. We selected this video because of its high-definition images, positively reinforcing music, and emotionally calming content. Patients watched the video on a 14-in high-definition computer screen placed in easy viewing proximity on a bedside or chairside table. We ran the video for its first 15 minutes—the same duration as the VR intervention.

### Study Procedures

Patients in both study periods were informed that researchers were testing the effect of a distraction experience on the perception of pain. Because it was important for the research staff to exhibit equipoise when describing the potential benefits of the study intervention (ie, VR in cohort 1, nature video in cohort 2), we prepared a script that used neutral language regarding the study intervention. Once consented, patients rated their current pain using a standard 11-point numeric rating scale (NRS), ranging from 0 (no pain) to 10 (“the worst pain of your life”). The NRS is a validated measure of pain widely employed in clinical practice based on its ease of use, high compliance rates, and responsiveness to detect meaningful changes in pain [22]. Although predominantly tested for somatic pain, we have previously demonstrated psychometric validity of the NRS for visceral pain [23]. Patients in both groups repeated the NRS assessment 2 minutes after completion of the intervention. In addition, because VR has potential for adverse events, we evaluated for dizziness, vertigo, nausea, vomiting, and seizures. We also measured changes in blood pressure and heart rate in the VR group.

### Statistical Analysis and Sample Size

We calculated descriptive statistics for key demographic and clinical characteristics between groups, including age, sex, race and ethnicity, primary reason for hospitalization, and baseline pain scores. We performed bivariate analysis to evaluate for significant differences between groups, including two-sample *t* tests for continuous parametric variables and chi-square tests for categorical variables.

We next compared pre- and postintervention pain scores within subjects (using paired *t* tests) and then compared difference-in-difference (DID) pain scores between cohorts (using the rank sum test given nonparametric DID distributions). In addition, we classified each individual patient as a responder or nonresponder using the criterion standard of achieving an effect size of ≥0.5 standard deviation on the pain scale, a “medium” effect size using the rule of Cohen, and a value corresponding to the minimum clinically important difference (MCID) using the rule of Norman [24]. We compared the proportion responding between groups using chi-square test and calculated the number needed to treat (NNT) between groups.

Because the study used a mixed factorial design, we used a repeated-measures analysis of variance (ANOVA), which incorporated both a between-subjects and within-subject factor (pretest-posttest). The *F* ratio of interest in the analysis was the interaction between the 2 factors, representing the treatment main effect. After estimation, we calculated eta squared (η^2^), which can be interpreted as how much of the variation in the sample can be explained by the interaction.

Finally, to adjust for potential differences in patient characteristics between groups, we performed multivariable linear regression analysis to test the independent effect of VR on pain reduction, adjusting for demographic and clinical variables. To support a regression model with 5 covariates, and assuming at least 20 subjects per covariate, we required a total sample size of 100 patients. All analyses were conducted using Stata 14 (StataCorp).

### Approval

The Cedars-Sinai Institutional Review Board approved this study (Cedars IRB Pro00039751).

## Results

### Patient Characteristics

There were 50 patients in each group (N=100). Table 1 provides baseline demographic and clinical characteristics. There were no significant differences between groups for age, sex, race, or ethnicity. The reasons for admission between groups were similar except for the proportion admitted for pulmonary reasons (higher in control group) and orthopedic reasons (higher in VR group). The mean baseline pain score was the same (5.4 points) in both groups.

**Table 1:** Patient characteristics.

Patient characteristics		VR^a^ group (n=50), n (%)	Controls (n=50), n (%)	*P* value^b^
Age in years, mean (SD)		54.48 (17.9)	47.7 (15.2)	.10
Sex, female		30 (60)	23 (46)	
Sex, male		20 (40)	27 (54)	.16
**Race/ethnicity**				.73
	Non-Hispanic white	25 (50)	26 (52)	.94
	Black	11 (22)	14 (28)	.55
	Asian	2 (4)	2 (4)	>.99
	Hispanic	10 (20)	8 (16)	.64
	Other	2 (4)	0	.15
**Reason for hospitalization**				.007
	Gastrointestinal	9 (18)	19 (38)	.08
	Cardiac	8 (16)	3 (6)	.17
	Pain control	7 (14)	2 (4)	.91
	Infectious disease	6 (12)	4 (8)	.08
	Hematological/oncological	1 (2)	4 (8)	.25
	Neurological	1 (2)	2 (4)	.58
	Postsurgical	8 (16)	4 (8)	.27
	Pulmonary	4 (8)	0	.04
	Orthopedic	0	6 (12)	.01
	Other	6 (12)	6 (12)	>.99

^a^VR: virtual reality.

^b^We used *t* tests for continuous variable bivariate analyses and chi-square tests for categorical analyses (when differences were found, a test of proportions was used).

### Difference in Pain Scores

When focusing on within-subject changes in pain, there was a significant drop in pain in both the patients in the VR group (pre-VR mean 5.4, SD 2.6; post-VR mean 4.1, SD 2.7; delta=1.3; *P*<.001; percent reduction=24%) and the control patients (preintervention mean 5.4, SD 2.6; postintervention mean 4.8, SD 2.7; delta=0.6; *P*<.001; percent reduction=13.2%), with a larger drop in the VR group than controls (Table 2). When comparing *between* groups, the DID of −0.7 points was highly significant in favor of VR (*P*=.008). Using a binary responder definition of a ≥0.5 standard deviation drop in pain, there was a higher proportion of responders in the VR group (65%) versus the control group (40%; *P*=.01, absolute difference=25%, NNT=4). In the repeated-measures ANOVA, results showed that VR elicited a statistically significant difference in pain scores following treatment, *F*_1,97_=7.45, *P*<.001. The calculated η^2^ was .07, which Cohen considers equivalent to an effect size slightly greater than medium. In multivariable regression analysis adjusting for age, race, ethnicity, sex, and reason for hospitalization, VR remained a significant predictor of pain reduction (beta coefficient=−0.65 point, 95% CI −1.3 to 0, *P*=.05). There were no differences in the effect of VR by age, race, ethnicity, sex, or reason for hospitalization.

**Table 2:** Results on pain.

Group	Preintervention pain score, mean (SD)	Postintervention pain score, mean (SD)	Difference (% drop)	*P* value
Virtual reality	5.4 (2.6)	4.1 (2.7)	1.3 (24)	<.001
Control	5.4 (2.6)	4.8 (2.7)	0.6 (13.2)	<.001
Between-group difference			0.7	.008

### Virtual Reality Adverse Event Monitoring

All patients in the VR group completed the Pain RelieVR experience in its entirety and reported no adverse outcomes. There was no statistically significant difference between pre-VR and post-VR systolic blood pressure, diastolic blood pressure, and heart rate measurements (*P*>.05; Table 3).

**Table 3:** Effect of virtual reality on blood pressure and heart rate.

Outcome		Pre-VR^a^	Post-VR	*P* value
**Systolic blood pressure, mm Hg (n=50)**				.32
	Mean	119.8	118.6	
	SD	17.4	16.9	
	Range	83-175	84-152	
	95% CI	115.4-124.2	114.37-122.93	
**Diastolic blood pressure, mm Hg (n=50)**				.18
	Mean	66.8	69.5	
	SD	11.7	11.2	
	Range	46-99	48-97	
	95% CI	63.85-69.75	65.7-71.4	
**Heart rate, beats per minute (n=50)**				.88
	Mean	77.9	77.8	
	SD	16.2	16.8	
	Range	46-118	49-122	
	95% CI	73.82-82.04	73.53-82.03	

^a^VR: virtual reality.

## Discussion

Although VR has been studied in a variety of conditions including wound care, rehabilitation, and anxiety, its effectiveness for managing pain in hospitalized patients has not been fully examined. In this study, we found that use of a 15-minute VR intervention in a diverse group of hospitalized patients resulted in statistically significant and clinically relevant (NNT=4) improvements in pain versus a control distraction video without triggering adverse events or altering vital signs. These results indicate that VR may be an effective adjunctive therapy to complement traditional pain management protocols in hospitalized patients.

Whereas previous VR research has traditionally focused on specific types of pain [6-10,12,25,26], our study is unique for evaluating VR across a wide range of somatic and visceral pain conditions. In multivariable regression analysis, we found the effect of VR was independent of the reason for hospitalization or primary cause of pain, suggesting that VR has benefits across wide groups of inpatients. Because this study is focused on a single pain distraction visualization, future research should evaluate whether and how to tailor VR content for specific pain syndromes, as this may have incremental benefits over a single, generic VR intervention. Similarly, future research should investigate active VR interventions, such as mindful meditation visualizations, in addition to passive distraction experiences. Nonetheless, the finding that a single intervention improved pain across diverse conditions suggests a common mechanism for the pain benefits of VR.

It remains unknown exactly how VR works to reduce pain perception across conditions. Most proposed mechanisms attribute the benefit to simple distraction [6]. When the mind is deeply engaged in an immersive experience, it becomes difficult, if not impossible, to perceive stimuli outside of the field of attention [27]. By “hijacking” the auditory, visual, and proprioception senses, VR is thought to create an immersive distraction that restricts the mind from processing pain [6]. Additional research should evaluate the neurobiological mechanisms of VR across pain conditions and measure whether its benefits in hospitalized patients, in particular, extend beyond the immediate VR treatment period.

Our study has several important limitations. First, although we compared results between 2 well-characterized groups in this early phase VR study, this was not a randomized controlled trial. Nonetheless, we performed multivariable regression analysis to adjust for variations between groups and still found that exposure to VR was a significant predictor of reduced pain. Future research should randomize patients in a larger, prospective comparison trial. Second, the VR intervention was only 15 minutes long and included only one visualization; it is possible that pain may rebound after VR and/or longer-term benefits require more sustained and repeated exposure to varying content. Future research should evaluate the effect of altering the duration, intensity, frequency, and content of VR compared with control conditions. Third, because this was a onetime intervention we did not measure the impact of VR on use of pain medications, hospital length of stay, or postdischarge satisfaction scores. Nonetheless, this study is, to our knowledge, the first to measure the impact of VR on pain management versus a control condition among a diverse group of hospitalized patients. Fourth, it is impossible to know whether the greater effectiveness of the VR condition was due to presenting a 3D virtual environment (vs a 2D environment) or playing a highly involving, active game versus a passive distraction experience. It is not possible from this experimental design to determine definitively if the observed effect in pain reduction was due to the 3D versus 2D experience, active versus passive components, variations in visual and audio between conditions, or other attributes that measurably vary between arms. Our pragmatic trial is a first step along a path of additional investigations; future research should test other control conditions and visualization to understand whether there are unique benefits of the 3D VR experience over other control conditions. Finally, our protocol did not track the characteristics and reasons for patient ineligibility or refusal to use VR. However, our previous research found that many hospitalized patients are not eligible to use VR for various reasons, including active neurological symptoms, ongoing nausea or vomiting, injury to the face or neck, epilepsy, too frail or debilitated, or receiving mechanical ventilation. Moreover, we found that among those who are medically eligible to use VR, up to two-thirds are unwilling to try the technology, particularly older individuals. Taken together, these findings reveal barriers to widespread use of VR in hospitalized patients. Future research should study whether adoption rates are increasing and whether using VR is cost-effective for hospitals given variable patient uptake.

These results indicate that VR is an effective, safe, and feasible intervention to aid with pain management among diverse hospitalized patients. Larger randomized clinical trials are needed to better characterize its impact on longer-term pain perception, resource utilization, and postdischarge outcomes.

## Abbreviations

ANOVA: analysis of variance
DID: difference-in-difference
NNT: number needed to treat
NRS: numeric rating scale
3D: three-dimensional
2D: two-dimensional
VR: virtual reality
